# Age-related compositional and functional changes in the adult and breastfed buffalo rumen microbiome

**DOI:** 10.3389/fmicb.2024.1342804

**Published:** 2024-05-30

**Authors:** Yixue Xu, Tong Feng, Zixu Ding, Ling Li, Zhipeng Li, Kuiqing Cui, Weihua Chen, Hongping Pan, Peng Zhu, Qingyou Liu

**Affiliations:** ^1^State Key Laboratory for Conservation and Utilization of Subtropical Agro-Bioresources, Guangxi University, Nanning, China; ^2^Department of Bioinformatics and Systems Biology, Key Laboratory of Molecular Biophysics of the Ministry of Education, Hubei Key Laboratory of Bioinformatics and Molecular-imaging, Center for Artificial Biology, College of Life Science and Technology, Huazhong University of Science and Technology, Wuhan, China; ^3^Guangxi Key Laboratory of Buffalo Genetics, Nanning, China; ^4^Guangdong Provincial Key Laboratory of Animal Molecular Design and Precise Breeding, School of Life Science and Engineering, Foshan University, Foshan, China; ^5^Guangxi Key Laboratory of Beibu Gulf Marine Biodiversity Conservation, Beibu Gulf Marine Ecological Environment Field Observation and Research Station of Guangxi, Beibu Gulf University, Qinzhou, China

**Keywords:** buffalo, rumen, microorganisms, metagenomics, immunity, lignocellulose

## Abstract

**Introduction:**

The buffalo is an important domestic animal globally, providing milk, meat, and labor to more than 2 billion people in 67 countries. The rumen microorganisms of buffaloes play an indispensable role in enabling the healthy functionality and digestive function of buffalo organisms. Currently, there is a lack of clarity regarding the differences in the composition and function of rumen microorganisms among buffaloes at different growth stages.

**Methods:**

In this study, metagenomics sequencing technology was applied to examine the compositional and functional differences of rumen microorganisms in adult and breastfed buffaloes.

**Results:**

The results revealed that the rumen of adult buffaloes had significantly higher levels of the following dominant genera: Prevotella, UBA1711, RF16, Saccharofermentans, F23-D06, UBA1777, RUG472, and Methanobrevibacter_A. Interestingly, the dominant genera specific to the rumen of adult buffaloes showed a significant positive correlation (correlation>0.5, p-value<0.05) with both lignocellulose degradation-related carbohydrate-active enzymes (CAZymes) and immune signaling pathways activated by antigenic stimulation. The rumen of breastfed buffaloes had significantly higher levels of the following dominant genera: UBA629, CAG- 791, Selenomonas_C, Treponema_D, Succinivibrio, and RC9. Simultaneously, the rumen-dominant genera specific to breastfed buffaloes were significantly positively correlated (correlation>0.5, p-value<0.05) with CAZymes associated with lactose degradation, amino acid synthesis pathways, and antibiotic-producing pathways.

**Discussion:**

This indicates that rumen microorganisms in adult buffaloes are more engaged in lignocellulose degradation, whereas rumen microorganisms in breastfed buffaloes are more involved in lactose and amino acid degradation, as well as antibiotic production. In conclusion, these findings suggest a close relationship between differences in rumen microbes and the survival needs of buffaloes at different growth stages.

## Introduction

The buffalo, a globally significant domestic animal of great value to humans, like other ruminants, possesses a specialized four-chambered stomach consisting of the rumen, reticulum, omasum, and abomasum. This unique digestive system enables the transformation of low-nutritional-value forage into high-quality animal protein, allowing the buffalo to adapt to the life cycle of forage grass (Lei et al., 2018). The ability of buffaloes to digest plant feeds is primarily attributed to the function of the microbial community within their gastrointestinal tract (Mizrahi et al., 2021). The structure of the rumen microbial community is community-based and can be influenced by various factors, such as host species (Li et al., 2019), gender (Guo et al., 2022), genetic factors (Zhang et al., 2020), diet (Scharen et al., 2017), age, and early life microbial colonization (Furman et al., 2020). The rumen microbiota plays a pivotal role in the utilization of plant material by ruminants (Su et al., 2022). The efficiency of plant feed utilization by rumen flora has a direct impact on host animal feed efficiency (Li et al., 2019), energy acquisition efficiency (Sasson et al., 2017), methane emissions (Mizrahi et al., 2021), antibiotic resistance genes (Sabino et al., 2019), milk yield, and milk protein content (Xue et al., 2020).

Dietary changes have a significant effect on the digestive flora of animals (Scharen et al., 2017; Morais and Mizrahi, 2019; Furman et al., 2020; Cao et al., 2023). Previous studies have demonstrated that diet influences the development of rumen flora in dairy cows (Furman et al., 2020), and the composition of the gut flora of humans (Stewart et al., 2018), pigs (Lim et al., 2019), and goats (Cao et al., 2023) varies considerably between the period of breastfeeding and the composition of adulthood. This shift occurs as neonates consume solid foods and cease breastfeeding, leading to a microbiome that resembles a more adult-like state and exhibits increased microbial diversity. Beef cattle (Stewart et al., 2018), dairy cows (Xue et al., 2022), sheep (Lane et al., 2000), goats (Cao et al., 2023), and camels (Gharechahi et al., 2022) have been found to have important roles in rumen microbial functioning for the health of the host animal. However, little attention has been given to differences in rumen microbial composition and functioning between calf buffaloes during the breastfeeding stage and adult buffaloes.

In this study, we analyzed the rumen microbial metagenomic data from 18 breastfed buffaloes and 29 adult female buffaloes fed with feed. We found that adult buffaloes had a higher abundance of genera (Prevotella, Saccharofermentans, Methanobrevibacter_A, UBA1711, RF16, F23-D06, UBA1777, and RUG472), lignocellulose degradation-related CAZymes (AA1, AA6, AA10, GH5, etc.), and antigenic stimulus-activated immune signaling pathways. In contrast, breastfed buffaloes had a higher abundance of genera (Selenomonas_C, Treponema_D, Succinivibrio, UBA629, CAG-791, and RC9), lactose degradation-related CAZymes (GH35, GH42, etc.), amino acid synthesis pathways, and antibiotic production pathways.

## Materials and methods

### Sample collection

A total of 47 samples were collected for metagenome sequencing. Among these samples, 29 were obtained from adult female buffaloes fed with feed (90 ± 36sd months), while 18 were obtained from breastfed buffaloes (<1 month). To ensure diversity, adult buffaloes with no history of intestinal disease and calves in the breastfeeding period were individually confined in cleaned pens. After a 12-h fasting period, rumen samples were collected using a stomach tube. All samples were immediately frozen in liquid nitrogen and stored at −80°C until DNA extraction. Most samples were processed for DNA extraction within a week of collection.

### DNA extraction, library construction, and metagenomics sequencing

Three grams of each sample were used for DNA extraction. DNA was extracted using a mini-bead beater (BioSpec Products; Bartlesville, United States), followed by phenol–chloroform extraction through a bead-beating method. After precipitation with ethanol, the pellets were suspended in 50 μL of Tris–EDTA buffer. DNA quantification was performed using a NanoPhotometer® (IMPLEN, CA, USA) after staining with a Qubit® 2.0 Fluorometer (Life Technologies, CA, USA). DNA samples were stored at −80°C until they were further processed. We followed the TruSeq DNA sample preparation guide (Illumina, 15,026,486 Rev. C) method and procedure, using 500 ng DNA as the template. We selected qualified libraries for paired-end sequencing on the Illumina NovaSeq 6,000 platform, with a read length of 150 base pairs (PE150).

### Quality control and removal of host- and food-associated genomes

The paired-end raw sequencing reads were first trimmed using Trimmomatic (Bolger et al., 2014) (v.0.35) to eliminate vectors and low-quality bases. Sequences longer than 110 bases and with an average base quality greater than 30 after trimming were retained for subsequent analysis. To eliminate potential reads from the host or food, genomic sequences of buffalo (Luo et al., 2020), *Glycine max* (Shen et al., 2019), *Zea mays* (Jiao et al., 2017), and *Medicago truncatula* (Tang et al., 2014) were downloaded from the National Center for Biotechnology Information (NCBI) and used as references in Bowtie2 (Langmead and Salzberg, 2012) (v.2.3.3) analysis with the options “-p 10-very-sensitive”. Reads that aligned concordantly with references were removed as contamination.

### Annotation and functional characterization of MAG-encoded proteins

To enhance the accuracy of our results, we used the gut microbiota genome catalog of the buffalo species from a prior study as a reference (Tong et al., 2022). For predicting MAG-encoded proteins, we utilized Prodigal (Hyatt et al., 2010) (v.2.6.3). Additionally, we used CD-HIT (Fu et al., 2012) (v.4.8.1) to cluster the predicted proteins, employing the following options: “-c 0.95 -n 10 -d 0 -M 16000 -T 8”. These non-duplicate proteins were further scrutinized against the CAZy database through the usage of dbCAN2 (Zhang et al., 2018) and the EggNOG database using eggnog-mapper (v.4.5) (Huerta-Cepas et al., 2016). For the KEGG annotation, we use the metagenome pipeline (Liu et al., 2021) designed by Liu et al. (2023), in conjunction with its requisite common software, script files, and database annotation files (EasyMicrobiome).

### Identification of differential taxa between groups

To establish the microbial strains exhibiting marked differences in abundance among the selected sample groups, we initially evaluated the relative redundancy of the MAGs in each sample. We subsequently mapped the clean reads from each sample to all MAGs and calculated the proportional abundance of a given MAG as a percentage of the total number of reads mapped to all MAGs in that sample. Therefore, the total relative abundance of all MAGs in each sample invariably amounts to 100%. Relative abundances at higher taxonomic levels, including genera, families, and orders, were determined by summing the abundances of their respective daughter clades based on the GTDB-TK phylogenetic tree.

The LEfSe tool (Segata et al., 2011) was utilized to identify differential taxa among groups of samples, selecting those with LDA scores >2 as the significant taxa. The statistical significance of the differential taxa abundance between groups was revealed using the Wilcoxon test. Subsequently, we picked taxa that have a crucial role in the physiological functions linked with the context of grouping.

### Statistics

The alpha diversity of the microbial community was obtained using the Shannon index obtained from the R package vegan analysis, and the beta diversity was obtained from non-metric multidimensional scaling (NMDS) analysis using the metaMDS function of the R package vegan. Spearman’s rank correlation was used for all correlation analyses, with R > 0.5 or < −0.5 being considered strong correlations and *p*-values <0.05 being considered significant.

## Results

### Structure of microbial communities in the rumen of adult and breastfed buffaloes

**Figure 1:**
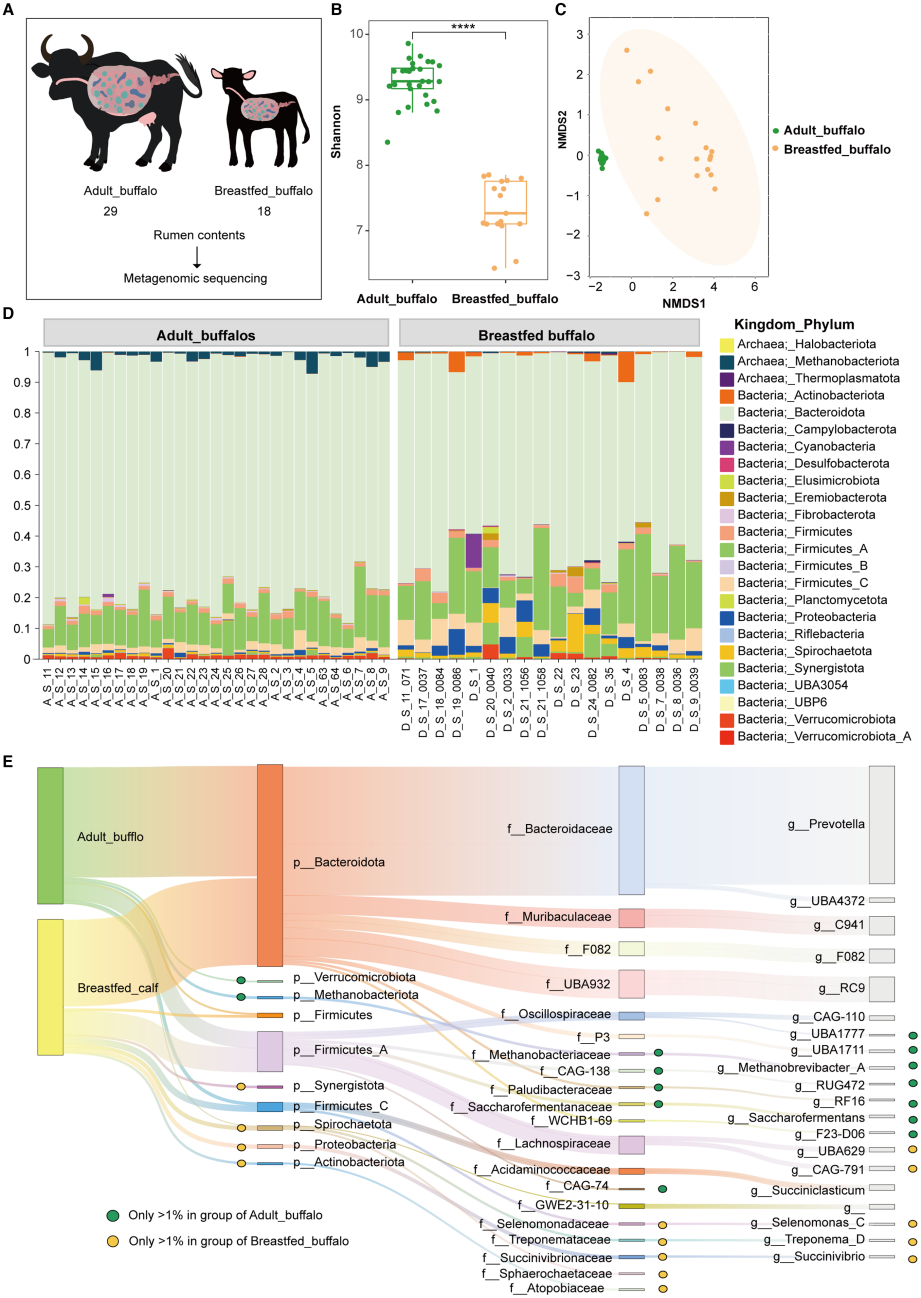
Compositional analysis of rumen microorganisms in adult and breastfed buffaloes. **(A)** Experimental design, macro genomic analysis of rumen contents of adult female buffaloes fed with feed (29), and breastfed buffaloes (18). **(B)** Shannon index. **(C)** NMDS analysis. **(D)** Percentage stacking of the phylum of rumen microorganisms in adult and breastfed buffaloes. **(E)** Sankey diagram of dominant rumen communities in adult and breastfed buffaloes. The diagram shows the dominant genera and the families and phyla to which they belong that are greater than 1% in the rumen of adult and breastfed buffaloes. The green dots represent that the genus, family, and phylum are greater than 1% in adult buffaloes only, the yellow dots represent that the genus, family, and phylum are greater than 1% in breastfed buffaloes only, and the unspecialized markings indicate that it is greater than 1% in both groups.

In this investigation, we collected rumen contents from 29 adult female buffaloes that were provided with feed, along with 18 breastfed buffaloes, followed by sequencing and bioinformatic analysis (Figure 1A). The Shannon index was higher in the adult buffalo group than in the breastfed buffalo group (Figure 1B, *p* < 0.0001), indicating that the diversity of rumen microorganisms was higher in adult buffaloes than in breastfed buffaloes. Adult buffaloes are exposed to a more complex environment than breastfed buffalo, with more time and opportunity for microbes to colonize. Forage is more difficult to digest compared to milk, so adult buffaloes need more microbes to assist in digesting forage. This results in a higher diversity of microbes colonizing the rumen of adult buffaloes. NMDS analyses showed that the rumen microbial communities of adult and breastfed buffaloes were significantly different (Figure 1C), with a higher degree of aggregation among microbial samples in adult buffaloes and a greater degree of disaggregation in breastfed calves, which was also seen in other species (Huang et al., 2018; Lim et al., 2019; Jin et al., 2021; Xiao et al., 2022).

We adopted the approach of Jin et al. (2022), designating that relative abundance greater than 1% is the dominant bacterial group. In this study, we counted the phyla, families, and genera of organisms in >1% of the rumen of adult and breastfed buffaloes. There were 6 phyla (97.50%), 14 families (93.05%), and 15 genera (84.87%) belonging to the dominant flora in the rumen of adult buffaloes. A total of 8 phyla (97.49%), 13 families (88.51%), and 13 genera (76.52%) were dominant in the rumen microflora of breastfed buffaloes (Supplementary Table S1). At the phylum level (Figure 1D), Bacteroidota (79.03, 64.96%), Firmicutes_A (11.75, 17.05%), Firmicutes_C (2.34, 4.48%), and Firmicutes (1.37, 1.93%) were present in both adult and breastfed buffalo. Methanobacteriota (1.85%) and Verrucomicrobiota (1.16%) were only dominant in adult buffalo, while Proteobacteria (2.86%), Spirochaetota (2.86%), Synergistota (1.72%), and Actinobacteriota (1.63%) were only dominant in breastfed buffalo. At the family level (Supplementary Figure S1B), Bacteroidaceae (56.28, 36.15%), UBA932(7.68, 12.52%), Muribaculaceae (4.82, 8.78%), F082 (4.54, 5.50%), Lachnospiraceae (2.61, 10.37%), Acidaminococcaceae (2.11, 2.27%), and Oscillospiraceae (2.41, 3.38%) were present in both adult and breastfed buffalo. Saccharofermentanaceae (2.21%), Acidaminococcaceae (2.11%), CAG-138(2.10%), P3(2.09%), Methanobacteriaceae (2.06%), Paludibacteraceae (1.64%), WCHB1-69 (1.62%), and CAG-74 (1.09%) were only dominant in adult buffalo, while Succinivibrionaceae (2.51%), Selenomonadaceae (1.68%), Treponemataceae (1.62%), Atopobiaceae (1.40%), Sphaerochaetaceae (1.23%), and P3 (1.08%) were only dominant in breastfed buffaloes.

The relative abundance of rumen flora in both adult buffaloes and breastfed buffaloes was greater than 1% in eight genera (Figure 1E), of which five, Prevotella (51.98,32.52%), RC9 (6.36,11.46%), C941 (4.70,8.58%), F082 (4.54,5.50%), and UBA4372 (1.94,1.28%), were the major contributors to Bacteroidota, while the remaining three, CAG-110(1.02, 2.19%), Succiniclasticum (2.09,1.95%), and unclassified GWE2-31-10_(1.68,1.71%), were derived from Firmicutes_A, Firmicutes_C, and Spirochaetota, respectively. There were seven genera with relative abundance greater than 1% in adult buffaloes only, of which four UBA1711 (1.91%), RF16 (1.40%), Saccharofermentans (1.96%), and F23-D06(1.19%) were derived from Bacteroidota, two UBA1777(1.05%), RUG472(1.19%) from Firmicutes_A, and Methanobrevibacter_A (1.85%) from Methanobacteriota. There were five genera with relative abundance greater than 1% in breastfed buffaloes only, two of them UBA629 (3.36%), CAG-791 (3.23%) were derived from Firmicutes_A, Selenomonas_C (1.55%), Treponema_D (1.62%), Succinivibrio (1.56%) from Firmicutes_C (4.48%), Spirochaetota (2.86%), and Proteobacteria (2.86%), respectively. In addition, the top 20 family-level and 30 genus-level microorganisms with the highest abundance per buffalo were enumerated (Supplementary Figures S3, S4).

### Analysis of differences in rumen microbiology between adult and breastfed buffaloes

**Figure 2:**
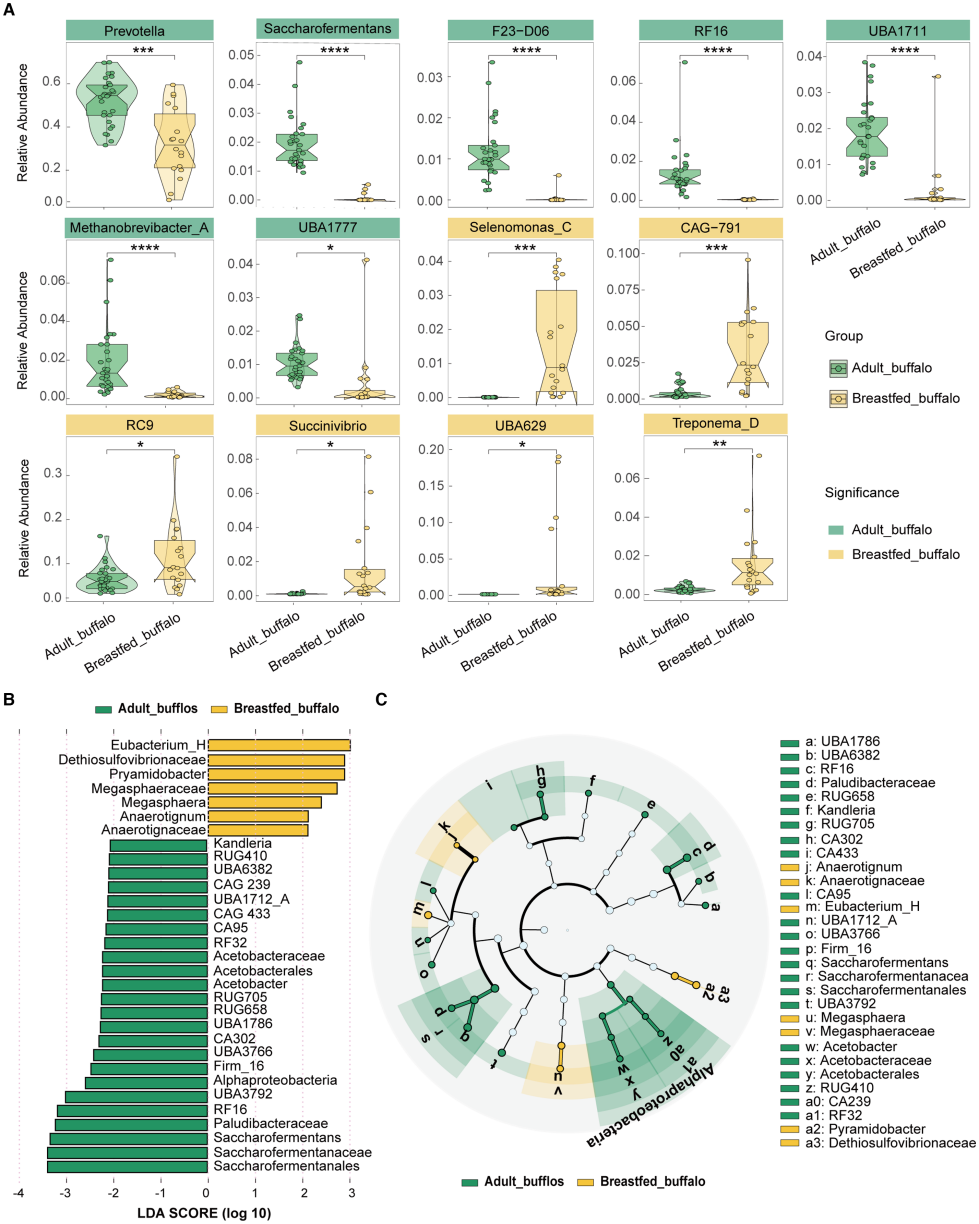
Composition and differential analysis of rumen microorganisms in adult and breastfed buffaloes. **(A)** Violin plots of the significant difference analysis of the dominant genus, with the color block where the genus name is located being green for significantly higher in adult buffalo and yellow for significantly higher in breastfed buffaloes. **(B)** Bar chart of the distribution of LDA values: demonstrates species that differed at LDA scores >2, with statistically different biomarkers, and the length of the histogram represents the magnitude of the influence of the significantly different species. **(C)** Species evolutionary branching diagram of the differing species, with circles radiating from inside to outside representing taxonomic levels from phylum to genus (or species). Each small circle at a different taxonomic level represents a taxon at that level, and the size of the circle diameter is proportional to the size of the relative abundance. Coloring principle: species with no significant differences are colored white, different species are colored following the group, yellow nodes indicate microbial taxa that play an important role in the breastfed buffalo group, and green nodes indicate microbial taxa that play an important role in the adult buffalo group. The full names of the species represented by the letters in the figure are shown in the legend.

Significant difference analysis was conducted for phylum, dominant family, and genus using STAMP software (Supplementary Figure S2). In adult buffaloes, seven phyla (all including Bacteroidota, Riflebacteria, UBP6, Methanobacteriota, UBA3054, Fibrobacterota, Planctomycetota), six dominant families (all including Bacteroidaceae, Saccharofermentanaceae, Methanobacteriaceae, Paludibacteraceae, CAG-138, WCHB1-69), and eight dominant genera (all including Prevotella, RC9, UBA1711, RF16, Saccharofermentans, F23-D06, UBA1777, RUG472) were found to be significantly higher (Figure 2A). While, breastfed buffaloes showed significantly higher levels of eight phyla (Proteobacteria, Spirochaetota, Desulfobacterota, Firmicutes_C, Firmicutes_B, Actinobacteriota, Campylobacterota, Firmicutes_A), eight dominant families (Lachnospiraceae, UBA932, Muribaculaceae, Succinivibrionaceae, Selenomonadaceae, Treponemataceae, Atopobiaceae, Sphaerochaetaceae), and six dominant genera (Selenomonas_C, Treponema_D, Succinivibrio, RC9, UBA629, CAG-791) (Figure 2A).LEfSe analysis of variance was conducted to obtain an overall view of the differential rumen microbes in adult and breastfed buffaloes. Linear Discriminant Analysis (LDA) was used to identify microorganisms that were statistically different between the adult buffalo and breastfed buffalo groups, with an LDA score >2 (Figure 2BC). Microorganisms that are higher in adult buffalo have Alphaproteobacteria, Saccharofermentanales, Acetobacterales, RF32, Saccharofermentanaceae, Paludibacteraceae, Acetobacteraceae, CAG-302, CAG-433, CAG-239, Saccharofermentans, RF16, UBA3792, g_Firm-16, UBA3766, UBA1786, RUG658, RUG705, Acetobacter, CAG-95, UBA1712_A, UBA6382, RUG410, Kandleria. Breastfed buffaloes had higher levels of Anaerotignaceae, Anaerotignum, Megasphaeraceae, Megasphaera, Pyramidobacter. In summary, genera with a relative abundance of over 1%, which exhibit no difference in both adult and breastfed buffalo (C941, F082, UBA4372, CAG-110, Succiniclasticum, unclassified GWE2-31-10_), may be the fundamental genera in the buffalo rumen. Significantly higher dominant genera in adult buffaloes (UBA1711, RF16, Saccharofermentans, F23-D06, UBA1777, RUG472, Methanobrevibacter_A) may be associated with degradation of lignocellulose, and significantly higher genera in breastfed buffaloes (UBA629, CAG-791, Selenomonas_C, Treponema_D, Succinivibrio) may be associated with degradation of high-protein, high-energy diets. These genera may have distinct roles in diverse dietary patterns at varying ages.

### Differences in the functional composition of rumen microorganisms in adult and breastfed buffaloes

**Figure 3:**
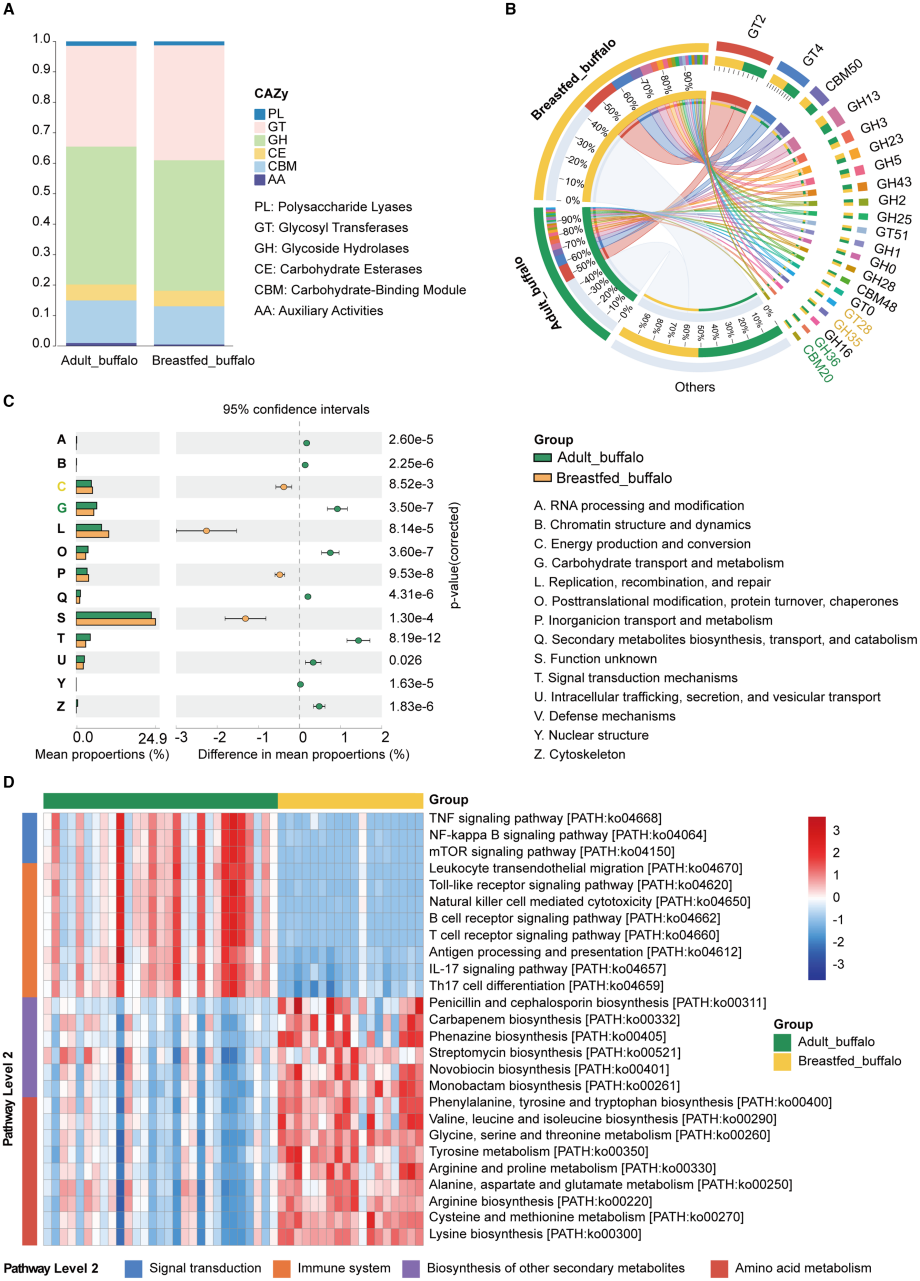
Functional composition and differential analysis of rumen microbes in adult and breastfed buffaloes. **(A)** Percentage CAZy stacking plot of rumen microbes of adult buffalo and breastfed calves. **(B)** CAZymes of rumen microbes greater than 1% in adult buffalo and breastfed calves. Green CAZymes numbers represent greater than 1% in adult buffalo only, yellow CAZymes numbers represent greater than 1% in breastfed buffalo only, and black CAZymes numbers indicate greater than 1% in both groups. **(C)** Analysis of rumen microbial COG function in adult and breastfed buffalo; on the left is a radar plot of the relative abundance of rumen microbial COG function in buffaloes and breastfed calves; and in the center is a STAMP difference analysis of rumen microbial COG in adult buffaloes and breastfed calves. The yellow color represents breastfed buffaloes, and the green color represents adult buffaloes. **(D)** Heatmap of differential KEGG tertiary pathways related to immunity in rumen microbes of adult buffalo and breastfed calves and the secondary pathways to which they belong.

In order to explore the functions of the excavated microbiota, we functionally annotated the predicted acquired genes for CAZyme, COG, and KEGG (Supplementary Table S1). Overall, glycoside hydrolases (GH) and glycosyl transferases (GT) were the major dominant CAZymes in both adult and breastfed buffaloes, with relatively higher GH, carbohydrate-binding module (CBM), and auxiliary activities (AAs) in adult buffaloes and higher relative abundance of GT in breastfed buffaloes (Figure 3A). We counted a high abundance of CAZymes (Figure 3B; Supplementary Table S1), and GH35 (Wang et al., 2020) with lactose degradation capacity had a relative abundance greater than 1% in breastfed buffaloes. In the COG database, significantly higher in breastfed buffaloes than in adult buffaloes C-Energy production and conversion (*p* < 0.001) enables the bacteria to access and utilise energy for survival and function. Adult buffalo rumen microorganisms had significantly higher G-Carbohydrate transport and metabolism to take up carbohydrates from the rumen and convert them into energy supply and metabolites (Figure 3C; Supplementary Table 1).

We also compared the KEGG database (Figure 3D; Supplementary Table S1). We found that amino acid metabolic pathways including alanine, aspartate, and glutamate metabolism; glycine, serine, and threonine metabolism; cysteine and methionine metabolism; valine, leucine, and isoleucine degradation; valine, leucine, and isoleucine biosynthesis; lysine biosynthesis; arginine and proline metabolism; tyrosine metabolism; and phenylalanine, tyrosine, and tryptophan biosynthesis were significantly higher in breastfed buffaloes than in adult buffaloes in terms of nutrient utilization. In immunity (Figure 3D; Supplementary Table S1), pathways related to antibiotic synthesis (carbapenem biosynthesis, monobactam biosynthesis, novobiocin biosynthesis, penicillin, cephalosporin biosynthesis, phenazine biosynthesis, and streptomycin biosynthesis) were significantly higher in breastfed buffalo than in adult buffalo, and immune signaling pathways (Toll-like receptor signaling pathway, Toll-like receptor signaling pathway, natural killer cell-mediated cytotoxicity, mTOR signaling pathway, leukocyte transendothelial migration, antigen processing and presentation, B cell receptor signaling pathway, T cell receptor signaling pathway, IL-17 signaling pathway, and Th17 cell differentiation) were significantly higher in adult buffalo than in breastfed buffalo. Adult buffalo exhibited greater binding to immune functions than breastfed buffalo, while the latter displayed higher amino acid metabolism and antibiotic synthesis. These findings suggest that rumen microbes play varying roles in immunity between adult and breastfed buffalo.

Overall, in terms of nutrition, rumen microbes in adult buffaloes are proficient in degrading lignocellulose and carbohydrates, while rumen microbes in suckling buffaloes are adept at degrading lactose and generating energy and amino acid metabolism. Regarding immunity, adult buffalo microbes rely on established immune signaling pathways, while rumen microbes in suckling buffaloes rely on self-produced antibiotics to combat pathogenic microbes.

### Spearman’s correlation analysis of dominant microorganisms and functions in adult and breastfed buffaloes

**Figure 4:**
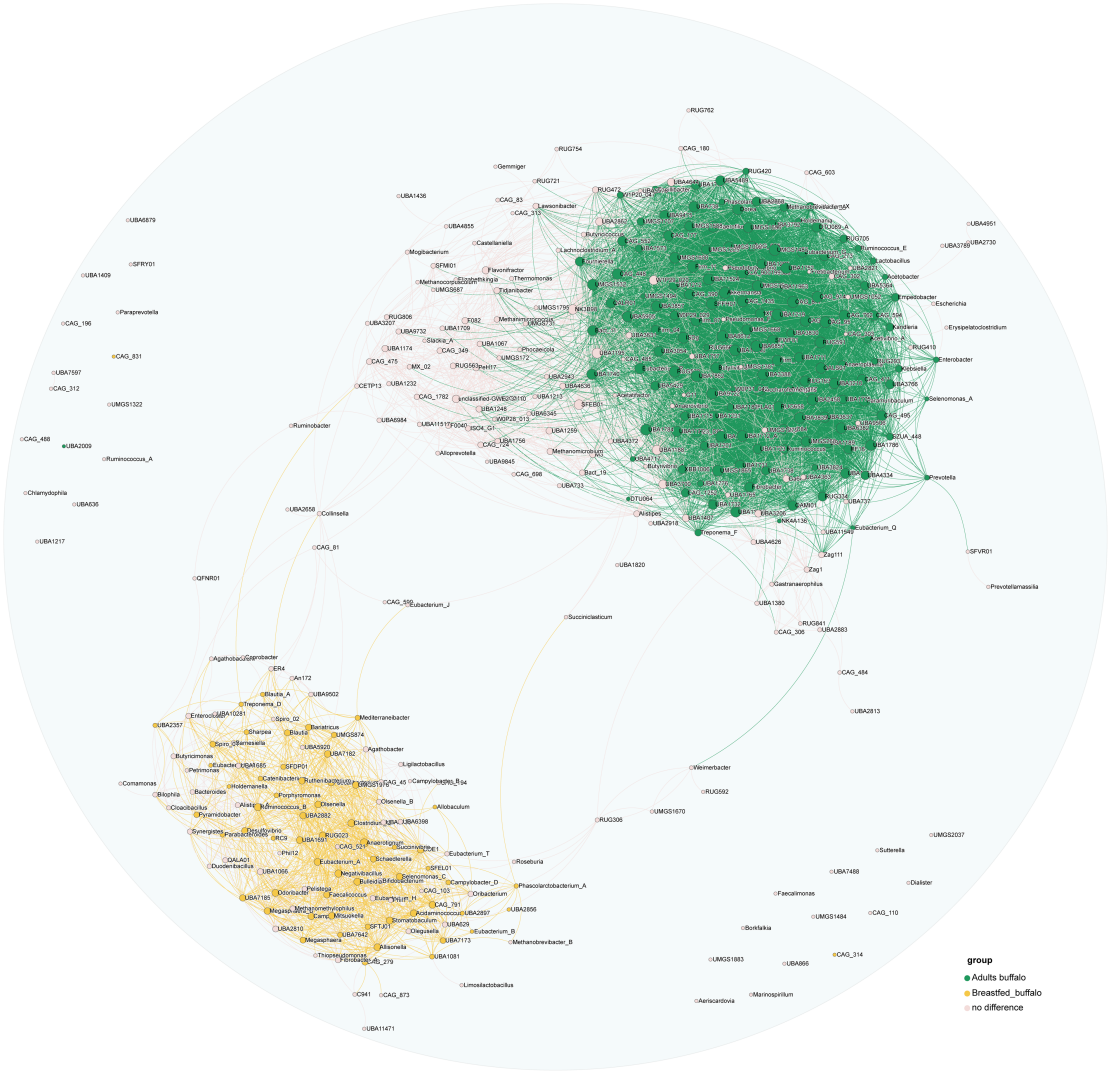
Plot of significant positive correlations between genera in the rumen of adult and breastfed buffaloes. The green dots represent all genera that were more abundant in adult buffaloes, the yellow dots represent all genera that were significantly more abundant in the rumen of breastfed buffaloes, the pink dots represent genera that did not differ between the two groups, and all the lines represent significant positive correlations between the two genera that are connected (correlation>0.5, *p*-value<0.05).

We further explored the potential microbiota functional relationship with an analysis of all identified genera to assess their correlations, and genera exhibiting significant positive correlations were visually represented on a co-occurrence diagram (correlation>0.5, *p*-value<0.05, Figure 4). We found a clear trend of segregation of significantly different genera between the two groups, with stronger aggregation of significantly higher genera (green nodes) in the rumen of adult buffaloes and stronger aggregation among significantly higher genera (yellow nodes) in the rumen of breastfed calves, suggesting that we have a community effect and symbiotic relationship between microorganisms in the rumen of buffaloes at different growth stages (Figure 4).

**Figure 5:**
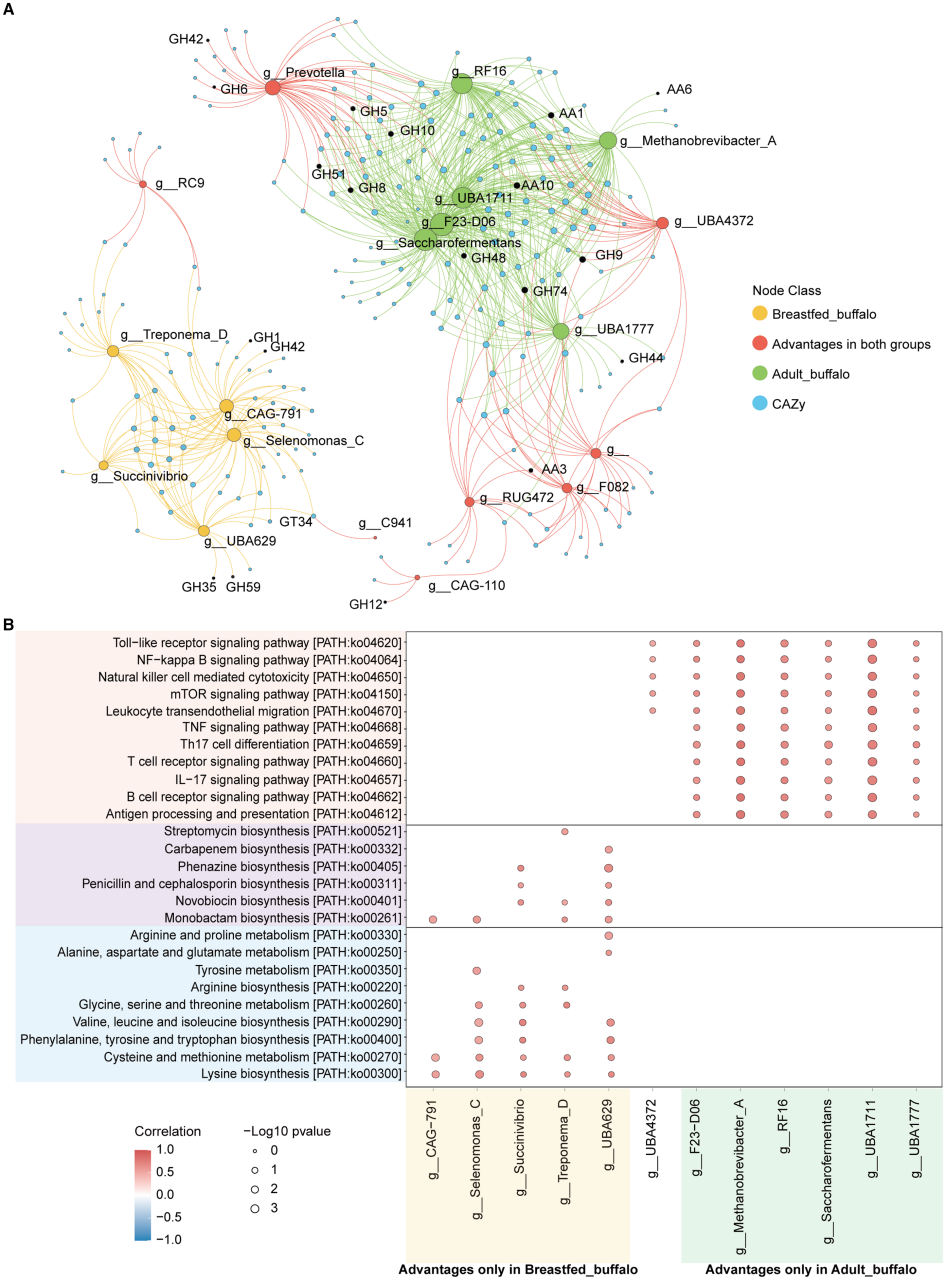
Correlation analysis of rumen microorganisms and function in adult and breastfed buffaloes. **(A)** Network diagram of significant positive correlation (correlation>0.5, *p*-value<0.05) between rumen microorganisms and CAZymes in adult and breastfed buffaloes. The green dots represent the dominant genera in adult buffaloes, the yellow dots represent the dominant genera in breastfed buffaloes, the red dots represent the co-dominant genera in both groups, and the blue dots represent the CAZymes. The green line connects the dominant genera specific to adult buffaloes with the CAZymes that are significantly positively correlated with them, and the yellow line connects the dominant genera specific to breastfed buffaloes with the CAZymes that are significantly positively correlated with them. CAZymes, the red lines connect the dominant genera, and CAZymes significantly positively correlated with both groups >1%. **(B)** Bubble heatmap of rumen microbial and immune-related KEGG tertiary pathway significantly positively correlated (correlation>0.5, *p*-value<0.05) in adult and breastfed buffaloes. Horizontal coordinates represent genera, and vertical coordinates are KEGG level 3 pathways. The size of the bubbles represents the level of significance; the smaller the *p*-value, the larger the bubbles, and the redder the color of the bubbles, the higher the correlation coefficient.

We correlated dominant genera and CAZymes and found that CAZymes associated with lignocellulose degradation (AA1, AA6, AA10, GH5, GH8, GH9, GH10, GH44, GH48, GH51, and GH74) were significantly positively correlated with dominant genera in adult buffaloes (correlation >0.5, *p*-value<0.05, Figure 5A), and CAZymes associated with lactose degradation (GH1, GH35, GH42, GH59, and GH39) were significantly positively correlated with dominant genera in breastfed buffaloes (correlation >0.5, *p*-value<0.05). This suggests that microorganisms in the rumen of adult and breastfed buffaloes have a clear preference for substrate digestion. A large proportion of CAZymes was significantly positively correlated both with dominant genera in the rumen of adult buffaloes and with dominant genera shared by both groups (Prevotella, UBA4372, RUG472, and F082). Many of these CAZymes were lignocellulose degradation-related; for example, AA3, GH6, and GH12 were significantly positively correlated with Prevotella and UBA4372. This implies that the rumen microorganisms of our breastfed buffaloes also have some lignocellulose degrading ability, and the pre-weaning colonization of rumen microorganisms provides sufficient preparation for the calves to feed on forage and pasture later on.

Then, we found out that pathways associated with amino acid metabolism, energy production, and antibiotic production were significantly positively correlated with the dominant genus of bacteria in the rumen of breastfed buffaloes. Interestingly, antibiotic production appeared to be dependent on amino acid metabolism and energy production. For example, the intermediate chorismate of phenylalanine, tyrosine, and tryptophan biosynthesis is required as an initial substance for phenazine biosynthesis, and both pathways are significantly positively correlated with Succinivibrio and UBA629. The dominant genera, specific to adult buffaloes, were significantly positively correlated with the previously mentioned immune-related pathways (correlation >0.5, *p*-value<0.05, Figure 5B). The positive correlation between UBA4372 (correlation >0.5, *p*-value<0.05, Figure 5B), which is the dominant genus in the rumen of both adult and breastfed buffaloes, and the immune signaling pathways (Toll-like receptor signaling pathway, Toll-like receptor signaling pathway, natural killer cell-mediated cytotoxicity, mTOR signaling pathway, and leukocyte transendothelial migration) that do not require antigenic stimulation suggests that UBA4372 may play an important role in the rumen immunity of buffaloes at different stages of growth.

Based on the above analysis, the rumen microorganisms of breastfed buffaloes have their own unique physiological functions, and the synthesis of amino acids and antibiotics is genus-specific. However, we did not find any genus that was significantly positively correlated with all metabolic pathways in buffaloes. We speculate that the rumen microorganisms of breastfed buffaloes may need to cooperate with each other to provide energy, produce and metabolize amino acids, and produce antimicrobial substances. This suggests that the nutritional requirements of breastfed buffaloes should be high in energy and protein, which is not only important for the growth and development of buffaloes but also for their resistance to diseases during the breastfeeding period.

## Discussion

In this study, we analyzed the microbiome data of rumen contents from 29 adult female buffaloes that were provided with feed, along with 18 breastfed buffaloes. The abundance (Shannon index) and similarity (NMDS) of rumen microorganisms were significantly higher in adult buffaloes than in breastfed buffaloes, and this pattern was also observed in dairy cows (Furman et al., 2020) and goats (Cao et al., 2023). This can be due to the fact that the microorganisms are still in the colonization stage at early life stages and are influenced by various factors such as the mode of delivery pregnancy diet (Lundgren et al., 2018), feeding mode (Stearns et al., 2017), and environment (Depner et al., 2020), so there is a wide variation within the group. Aging and highly similar diets contribute to richer and more similar rumen microorganisms in the rumen of adult buffaloes. This could be linked to the nutritional and immunological needs of adult and nursing buffaloes during this stage of their growth, as well as their strong association with the primary diet of adult and nursing buffaloes.

**Figure 6:**
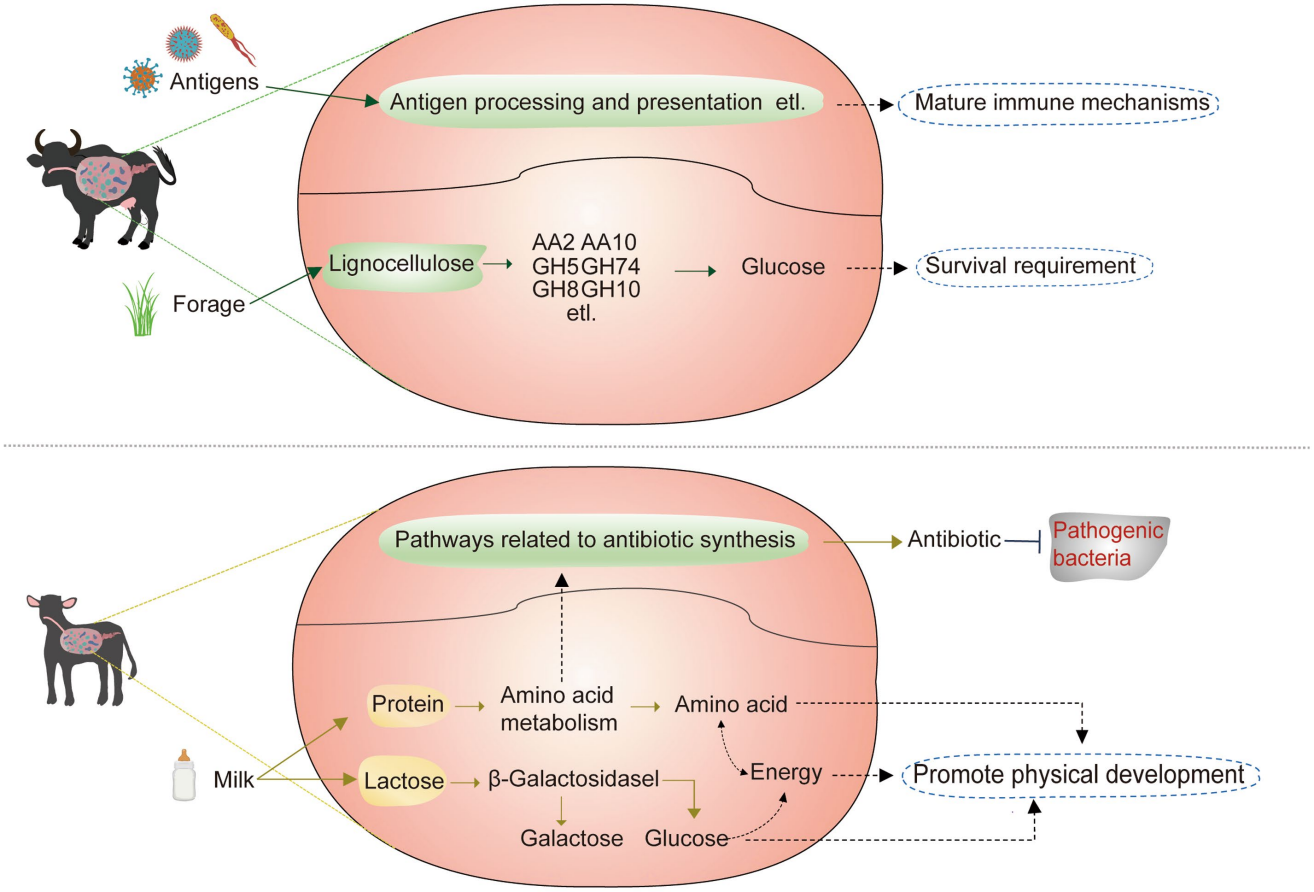
Physiological functions assumed for rumen execution in adult and breastfed buffaloes.

In terms of nutritional requirements, dietary differences have resulted in dominant rumen microbes specific to buffaloes at different growth stages. Significantly higher dominant microbes in the rumen of breastfed buffaloes were found to be positively correlated with lactose degradation-related CAZymes, amino acid metabolism pathways, and pathways of energy synthesis and utilization, and a low-fiber, high-energy, high-protein diet has been reported to elevate the levels of Succinivibrio (Tang et al., 2019) in abundance. Selenomonas_C, CAG-791, Treponema_D, RC9, and UBA629. We speculate that these five genera also have the potential to degrade high-energy, high-protein substrates such as milk. Significantly higher dominant microorganisms in the rumen of adult buffaloes were all significantly positively correlated with CAZymes associated with lignocellulose degradation (Figure 6), with Prevotella (Maus et al., 2020) and Saccharofermentans (Perea et al., 2017) reported to be associated with lignocellulose degradation. UBA1711, F23-D06, UBA1777, Methanobrevibacter_A, RF16, Prevotella, and Saccharofermentans have co-associated and lignocellulose degradation-related CAZymes, so we suggest that these five genera may have lignocellulose degradation potential.

In terms of immune requirements, a higher abundance of antibiotic synthesis pathways has been annotated in the rumen microbes of breastfed buffaloes. The products of these pathways, including penicillin, cephalosporin, carbapenem, monobactam, novobiocin, phenazine, and streptomycin (Majumdar and Kutzner, 1962; Bush and Bradford, 2016; May et al., 2017; Huigens et al., 2019; Armstrong et al., 2021) are antibiotics known to have activity against pathogenic microorganisms. The immune system of breastfed buffaloes is not well developed, and the microorganisms in the rumen have not formed a strong bond with the organism, so they rely on antibiotics produced by some genera in the rumen to defend themselves against pathogenic microorganisms. Adult buffalo rumen microbes are more closely linked to the body’s immune system, and higher abundances of antigen processing and presentation, the B cell receptor signaling pathway, the T cell receptor signaling pathway, the IL-17 signaling pathway, and Th17 cell differentiation contribute to the capture and presentation of antigens in the rumen (Kaufmann and Schaible, 2005) and stimulate the activation of immune cells and immune factors, resulting in a series of immune responses.

Remarkably, amino acid metabolic pathways significantly positively correlated with the dominant rumen microorganisms of breastfed buffaloes, which appeared to have a significant impact on antibiotic synthesis (Figure 6). The intermediate chorismate of phenylalanine, tyrosine, and tryptophan biosynthesis is required for phenazine biosynthesis. Additionally, the intermediate L-proline (C00148) of arginine and proline metabolism serves as the initial compound for novobiocin biosynthesis. All four pathways were significantly positively correlated with UBA629, while UBA629 was significantly positively correlated with the most antibiotic synthesis pathways, suggesting that UBA629 may be the main antibiotic-producing genus in the rumen of breastfed buffaloes. Arginine enhances streptomycin production (Majumdar and Kutzner, 1962), and in our results, we also found that streptomycin biosynthesis and arginine biosynthesis were both significantly positively correlated with Treponema_D. This suggests that our amino acid synthesis and metabolism are important for buffaloes during the breastfeeding period, in addition to maintaining their immunological development. Meanwhile, Luo et al. found that Treponema_D and goat immunity are positively correlated (Luo et al., 2022), thus confirming our view from the side. Therefore, breastfed buffaloes can be fed a high protein diet to enhance immunity and maintain their growth needs and should not be supplemented with antibiotics to avoid damaging the rumen environment of breastfed buffaloes.

In conclusion, the composition and function of rumen microbes differed significantly between adult feed-fed and breastfed buffaloes. Adult buffaloes exhibited more similar rumen microbial compositions, with specific dominant genera that possess greater abilities to degrade lignocellulose and carbohydrates, as well as established immune pathways. The rumen microbial composition of breastfed buffaloes was more different, with particular dominant genera exhibiting greater benefits in lactose degradation, amino acid metabolism, and antibiotic synthesis. The contrasting dietary patterns of adult and breastfed buffaloes gave rise to differing dominant rumen microbes, which meet the nutritional and immune necessities of buffaloes at diverse stages of growth.

## Conclusion

In our study, we explored the composition and function of rumen microorganisms in adult and breastfed buffaloes. We discovered distinctive differences in the community and function of these microorganisms. Rumen microorganisms in adult buffalo primarily contribute to lignocellulose degradation and exhibit close associations with immune responses when exposed to antigens. In contrast, rumen microbes in breastfed buffaloes focus on galactose conversion and synthesizing antibiotics to resist pathogens. These variations arise from differences in growth stages and diets, offering valuable insights into the gut microorganisms of domestic animals.

## Data availability statement

Publicly available datasets were analyzed in this study. This data can be found here: the raw sequencing data used in this study are available in the NCBI SRA database under accession code PRJNA656389. The 4960 strain-level MAG data used for species annotation in this study are available in the figshare database under access code 17000302.

## Ethics statement

The animal studies were approved by Experimental Animal Ethics Committee, College of Animal Science and Technology, Guangxi University. The studies were conducted in accordance with the local legislation and institutional requirements. Written informed consent was obtained from the owners for the participation of their animals in this study.

## Author contributions

YX: Visualization, Validation, Project administration, Methodology, Investigation, Formal analysis, Data curation, Conceptualization, Writing – review & editing, Writing – original draft. TF: Methodology, Formal analysis, Data curation, Writing – review & editing. ZD: Investigation, Formal analysis, Data curation, Writing – review & editing. LL: Resources, Writing – review & editing. ZL: Supervision, Writing – review & editing. KC: Supervision, Writing – review & editing. WC: Supervision, Writing – review & editing. HP: Supervision, Project administration, Writing – review & editing. PZ: Supervision, Funding acquisition, Writing – review & editing. QL: Supervision, Resources, Project administration, Methodology, Funding acquisition, Conceptualization, Writing – review & editing.

## Supplementary material

SUPPLEMENTARY FIGURE S1 Stacked plots of dominant rumen microorganisms in adult and breastfed buffaloes. **(A)** Phylum-level. **(B)** Family-level. **(C)** Genus-level.

SUPPLEMENTARY FIGURE S2 Differential microbiology in adult and breastfed buffaloes. **(A)** Phylum-level. **(B)** Family-level >1%.

SUPPLEMENTARY FIGURE S3 Percentage stacking of family of rumen microorganisms of adult and breastfed buffaloes.

SUPPLEMENTARY FIGURE S4 Percentage stacking of genus of rumen microorganisms of adult and breastfed buffaloes.

SUPPLEMENTARY FIGURE S5 Heatmap of the level 2 kegg pathway of differences between adult and breastfed buffaloes.

SUPPLEMENTARY FIGURE S6 Heatmap of correlation bubbles of dominant bacterial genera and KEGG class 3 pathways in adult and breastfed buffaloes.
