# Supplementary figures and images for: Age-related compositional and functional changes in the adult and breastfed buffalo rumen microbiome
Source: Front Microbiol. 2024 May 30;15:1342804. doi: 10.3389/fmicb.2024.1342804 (PMC11177756; doi:10.3389/fmicb.2024.1342804)

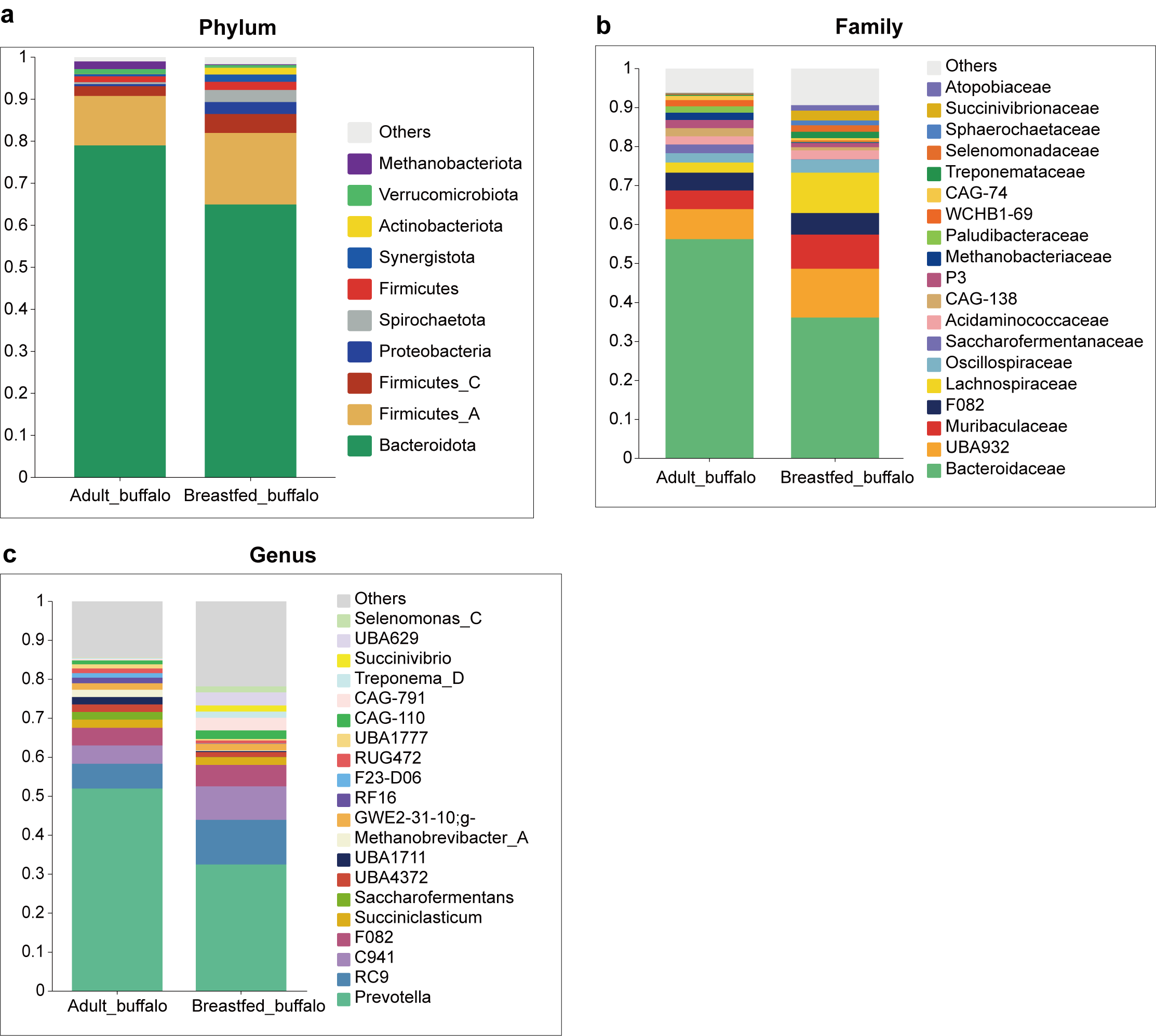

Supplement: SUPPLEMENTARY FIGURE S1 — Stacked plots of dominant rumen microorganisms in adult and breastfed buffaloes. (A) Phylum-level. (B) Family-level. (C) Genus-level. [file Image_1.TIF]

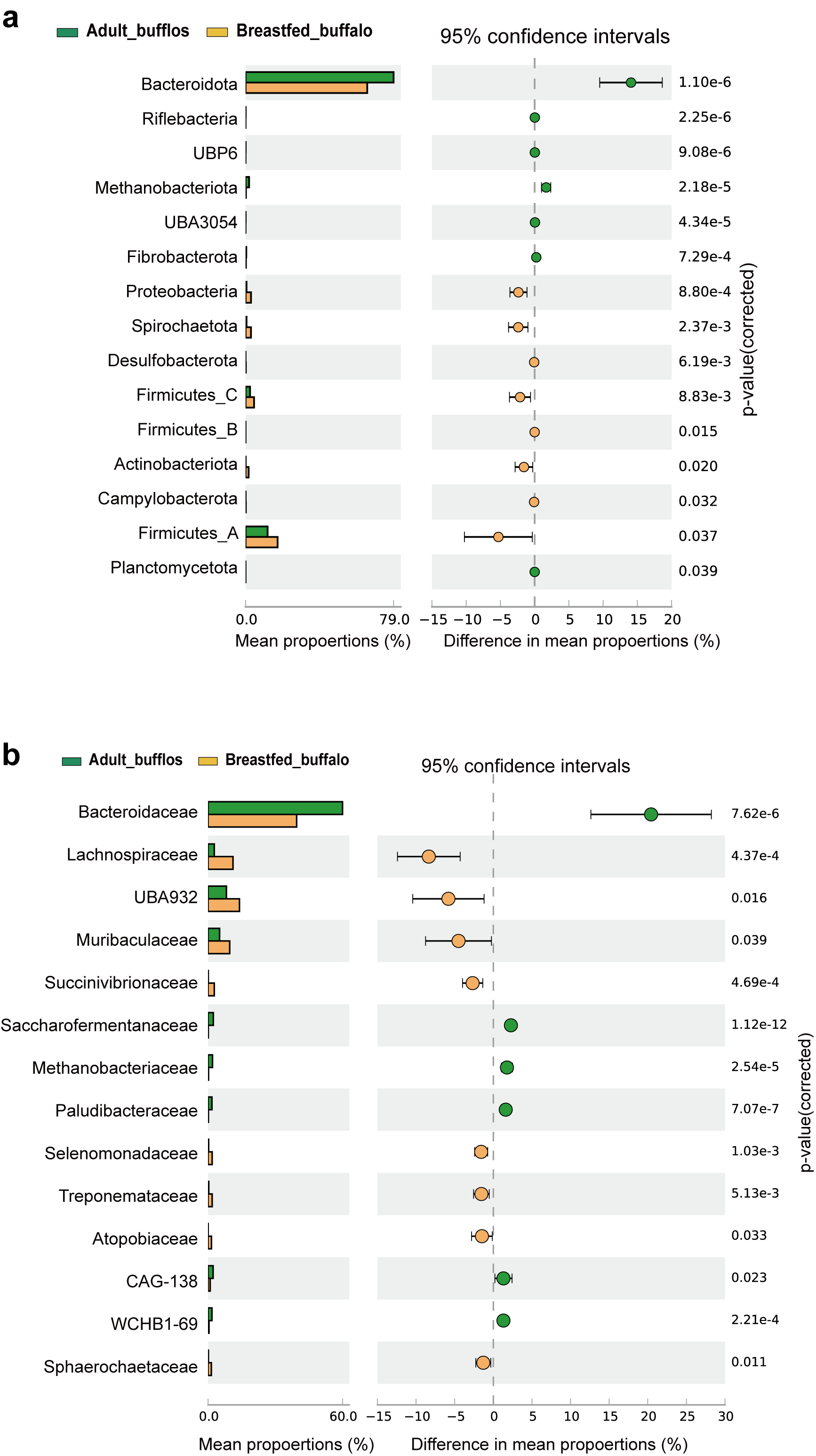

Supplement: SUPPLEMENTARY FIGURE S2 — Differential microbiology in adult and breastfed buffaloes. (A) Phylum-level. (B) Family-level >1%. [file Image_2.TIF]

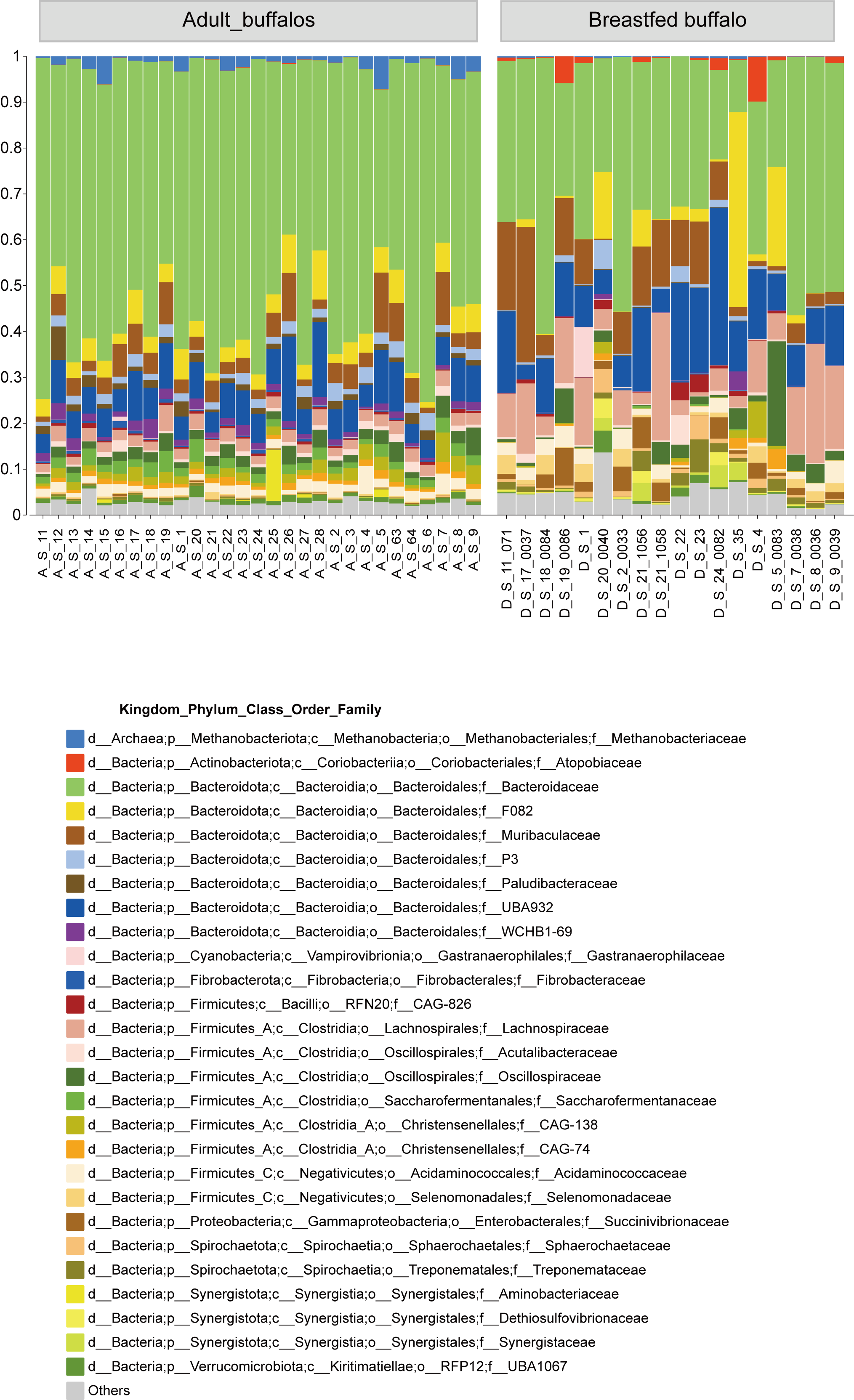

Supplement: SUPPLEMENTARY FIGURE S3 — Percentage stacking of family of rumen microorganisms of adult and breastfed buffaloes. [file Image_3.TIF]

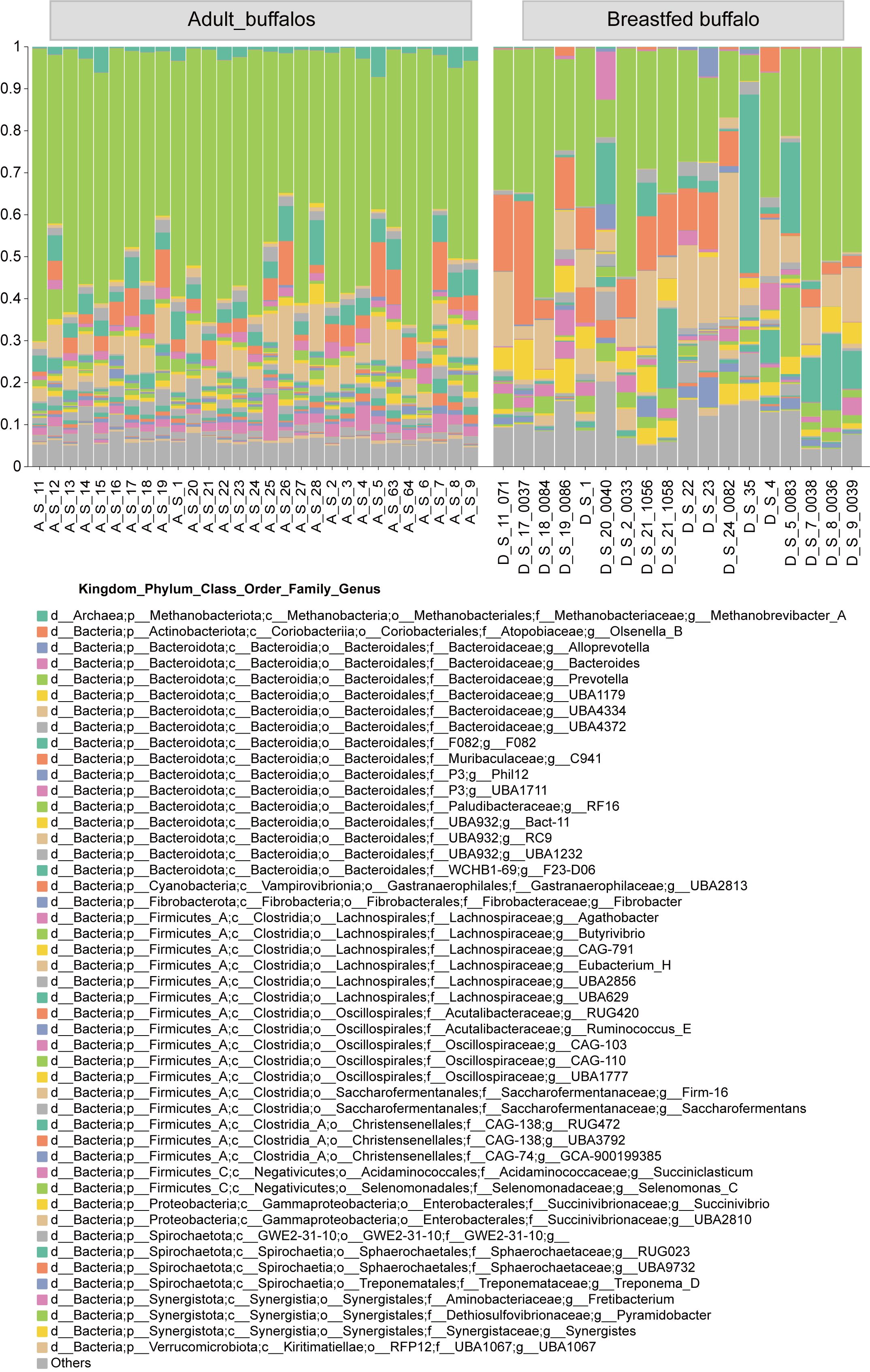

Supplement: SUPPLEMENTARY FIGURE S4 — Percentage stacking of genus of rumen microorganisms of adult and breastfed buffaloes. [file Image_4.TIF]

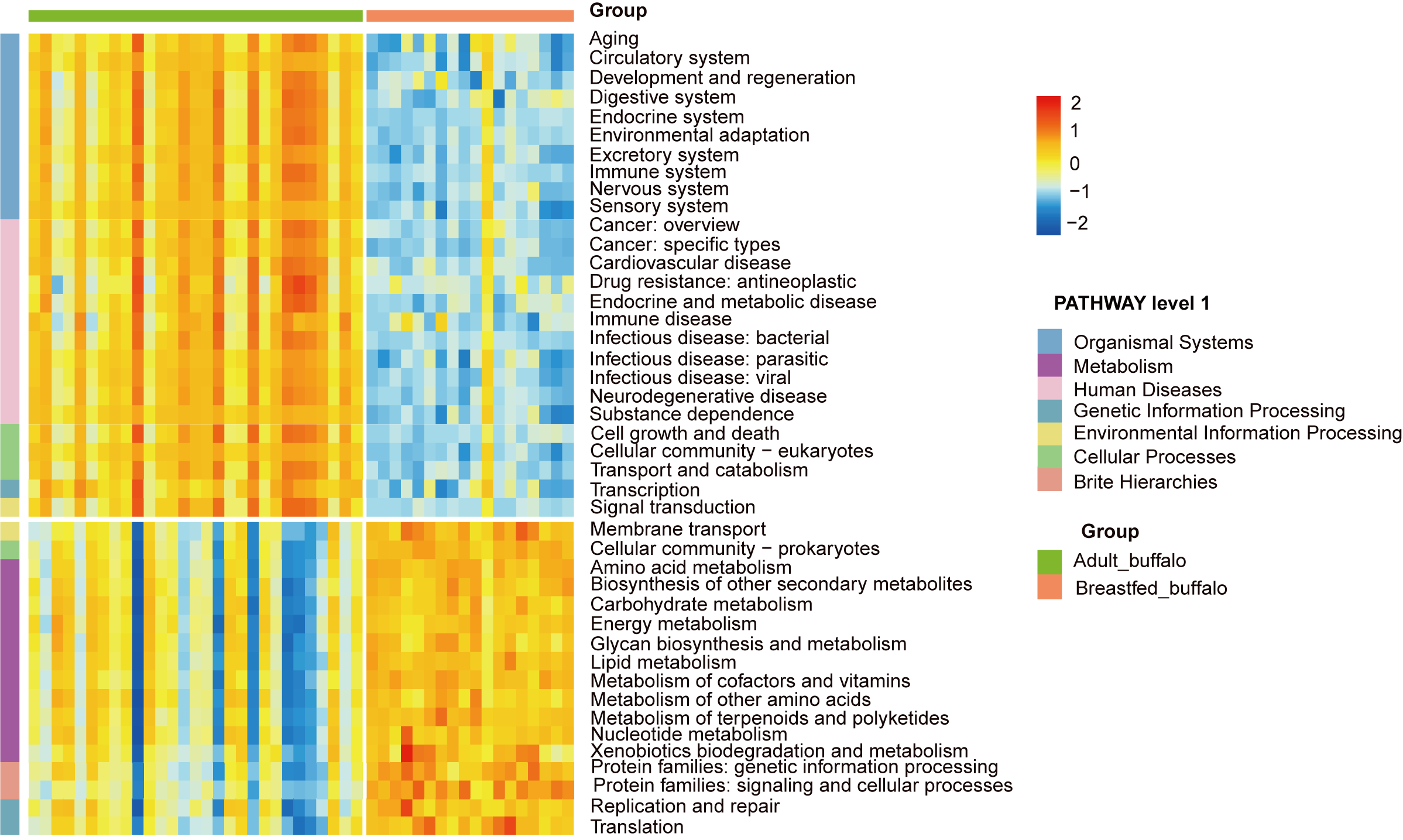

Supplement: SUPPLEMENTARY FIGURE S5 — Heatmap of the level 2 kegg pathway of differences between adult and breastfed buffaloes. [file Image_5.tif]

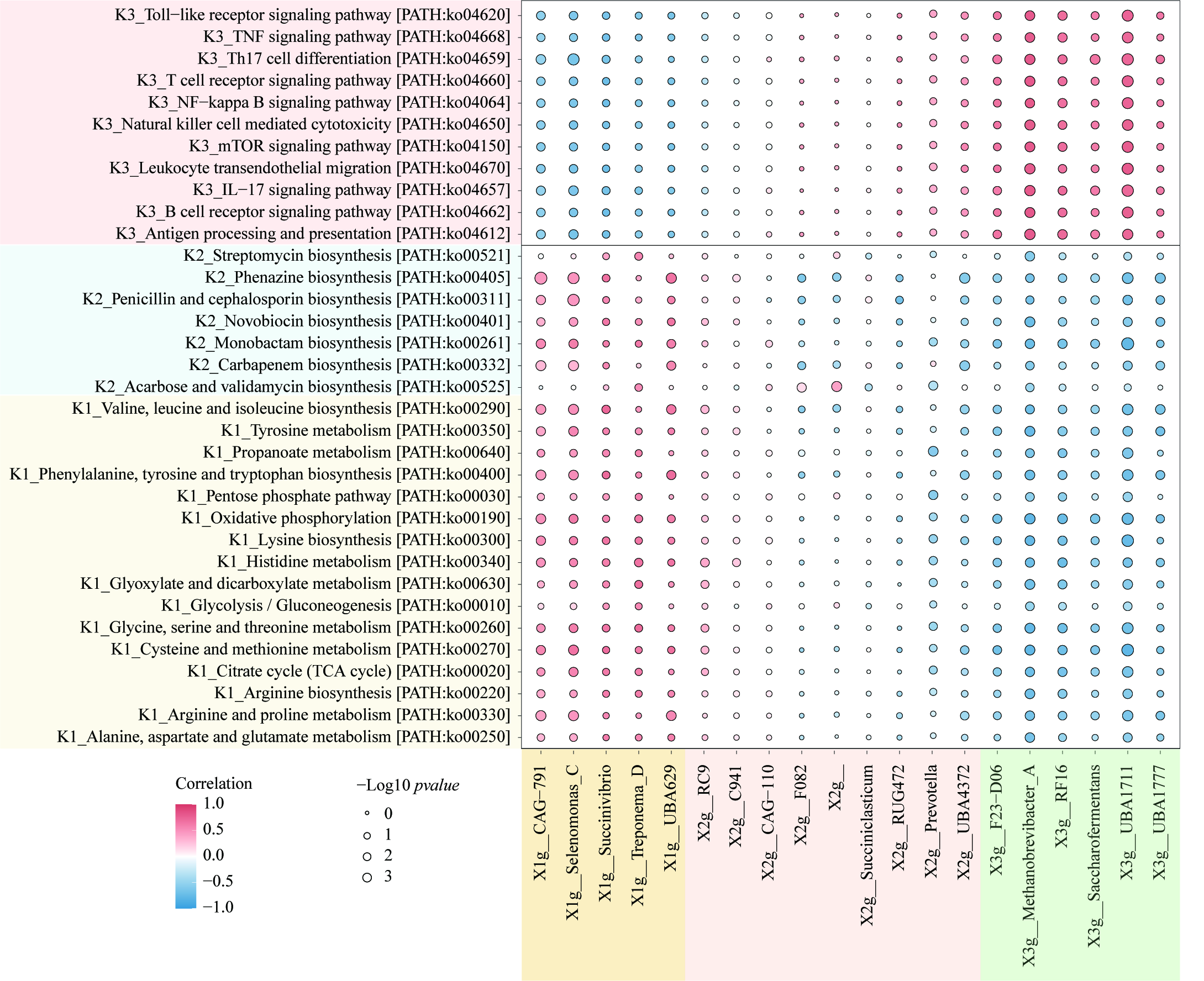

Supplement: SUPPLEMENTARY FIGURE S6 — Heatmap of correlation bubbles of dominant bacterial genera and KEGG class 3 pathways in adult and breastfed buffaloes. [file Image_6.TIF]
